# Flexible resource allocation during plant defense responses

**DOI:** 10.3389/fpls.2013.00324

**Published:** 2013-08-22

**Authors:** Jack C. Schultz, Heidi M. Appel, Abigail P. Ferrieri, Thomas M. Arnold

**Affiliations:** ^1^Christopher S. Bond Life Sciences Center, University of MissouriColumbia, MO, USA; ^2^Department of Molecular Ecology, Max Planck Institute for Chemical EcologyJena, Germany; ^3^Biochemistry and Molecular Biology Program, Department of Biology, Dickinson College, CarlislePA, USA

**Keywords:** carbon allocation, herbivory, nitrogen allocation, sequestration, plant defense

## Abstract

Plants are organisms composed of modules connected by xylem and phloem transport streams. Attack by both insects and pathogens elicits sometimes rapid defense responses in the attacked module. We have also known for some time that proteins are often reallocated away from pathogen-infected tissues, while the same infection sites may draw carbohydrates to them. This has been interpreted as a tug of war in which the plant withdraws critical resources to block microbial growth while the microbes attempt to acquire more resources. Sink-source regulated transport among modules of critical resources, particularly carbon and nitrogen, is also altered in response to attack. Insects and jasmonate can increase local sink strength, drawing carbohydrates that support defense production. Shortly after attack, carbohydrates may also be drawn to the root. The rate and direction of movement of photosynthate or signals in phloem in response to attack is subject to constraints that include branching, degree of connection among tissues, distance between sources and sinks, proximity, strength, and number of competing sinks, and phloem loading/unloading regulators. Movement of materials (e.g., amino acids, signals) to or from attack sites in xylem is less well understood but is partly driven by transpiration. The root is an influential sink and may regulate sink-source interactions and transport above and below ground as well as between the plant and the rhizosphere and nearby, connected plants. Research on resource translocation in response to pathogens or herbivores has focused on biochemical mechanisms; whole-plant research is needed to determine which, if any, of these plant behaviors actually influence plant fitness.

All parts of an individual plant are connected through the medium of its resource budget” Janzen (1973).

Janzen’s observation was intended to place the physiologically obvious in the context of plants as food for insects. He was making the point that as insects harvest material from one plant module, other modules are likely to feel the impact. We now know that those undamaged modules also supply materials for the production of defenses activated by insect attack and that some of the impact of herbivory on many plant organs involves investment in systemic defense production. How plants manage resource allocation in the face of conflicting demands has long intrigued ecologists and has shaped our thinking about plant evolution.

The view that organisms must make choices among investments from a limited resource budget rests comfortably within a framework familiar to both engineers (as “design constraints”) and economists (the “no free lunch” principle). It has a long history in biology as well. Darwin (1872) cited the “law of compensation or balancement of growth” of Saint-Hilaire and Goethe from the early nineteenth century, quoting Goethe:

“The budget of nature is fixed; but she is free to dispose of particular sums by an appropriation that may please her. In order to spend on one side, she is forced to economize on the other side” (Saint-Hilaire, 1818).

If a tradeoff exists between investment in defense and investment in other functions that influence fitness, then natural selection may favor the evolution of inducible defenses, whose expense occurs only when needed (Steppuhn and Baldwin, 2008). The necessary assumption that defense production has fitness costs has mixed support, largely because it is extremely difficult, if not impossible, to manipulate defense without confounding treatment effects. The ability to silence defense production or elicitation using transgenes is undoubtedly the best method we have available for pinpointing potential fitness costs of defense. A cost of producing chemical defenses has indeed been found in the few cases in which this method has been used (Schwachtje and Baldwin, 2008). As the ability to manipulate specific genes in more plant species becomes possible, we hope more such experiments will help clarify defense costs. It is possible that no experimental method will ever isolate cost of defense from other costs or influenced functions, since the gene networks involved in defense intersect with and influence networks regulating other fitness-related functions (Baldwin and Preston, 1999; Steppuhn and Baldwin, 2008). But broadly speaking, immunity to disease or herbivores is thought to incur fitness costs in all organisms, and this provides a justification for the evolution of inducible responses to attack.

## RESOURCE ALLOCATION IN DEFENSE

Responses to attack by insects or pathogens create a local need for resources to support defense. But herbivory, wounding and jasmonate decrease *local* photosynthetic rates (Creelman and Mullet, 1997; Beltrano et al., 1998; Hristova and Popova, 2002; Izaguirre et al., 2003; Schwachtje and Baldwin, 2008; Gomez et al., 2010) so that plants cannot add to their carbon pool in response to attack by increasing local carbon fixation. Similarly, evidence that nutrient uptake by roots is stimulated by herbivory is mixed, and uptake is frequently reduced. This means that the building blocks for defense production must be reallocated from existing pools or redirected from current “income” to support defense and provide resistance in response to attack. These materials must be translocated from distant tissues whenever locally available resources are not sufficient to support defense production, as in young, developing leaves (Arnold and Schultz, 2002; Arnold et al., 2004). For example, some have reported increases in photosynthetic activity in unattacked leaves following damage by defoliating herbivores (Nowak and Caldwell, 1984; Welter, 1989; Schwachtje and Baldwin, 2008), and have suggested that this may partially compensate for defoliation (Detling and Painter, 1983; Nowak and Caldwell, 1984). Shoots emerging to replace those lost to browsers, are especially dependent upon resource transport and are typically strong sinks (Iqbal et al., 2012); one result can be apparently increased plant productivity and increased resources for herbivorous insects (Nykänen and Koricheva, 2004). This review discusses how such a transport system might provide plant organs with the materials needed for defense in response to attack by herbivores (and microbes). It focuses on carbon and nitrogen as resources for defense, because those two elements have been the focus of defense theories and differ in how they are acquired and translocated.

The observation that plants comprise modules linked by access to and competition for resources means that resource needs for defense in one module may be met by translocation from other modules, and that two or more demanding modules may compete. Early studies attempted to link plant resource availability to plant defense focused primarily on “local” resources, already available at wound sites. More recently, we have come to understand that plants are a dynamic mosaic of resources and defensive substances. Observed changes in resource flow in response to insect or pathogen attack appear to be complex and sometimes even contradictory. We suggest that altered translocation of resources is a critical part of responses to insect and pathogen attack and needs to be incorporated into thinking about plant defense “strategy.”

## RESOURCES: CARBON

Plants fix about 560 Gt of atmospheric carbon per year (Jansson et al., 2010) and incorporate it into hundreds of thousands of molecular structures, many of which contain or are derived from sugars. For example, sugars and their direct products represent more than two-thirds of the dry weight of woody plants (Richardson et al., 2013). Plants also amass and store soluble sugars and starch as nonstructural carbohydrates (NSC) to support future respiration and growth as well as metabolic processes generating plant defenses (Chapin et al., 1990). The mass of stored NSCs can also be significant; for example, Würth et al. (2005) estimated that 8% of the living biomass of a tropical forest consisted of soluble sugars and starch. Stored NSCs provide plants with the ability to replace tissues lost to pests and other stresses. For example, temperate trees store enough NSCs to regrow their canopies four times (Millard and Grelet, 2010). Under most circumstances these pools are never fully depleted (see Millard and Grelet, 2010). For some species, cyclitols such as quinic and shikimic acids play a similar role as stored carbon banks (Kozlowski, 1992). While these compounds are the basis of growth and reproduction, many contribute directly or indirectly to the defense of plants against their enemies.

Carbon is also in demand when a plant tissue is called upon to defend itself. This can represent significant metabolic cost in terms of biosynthesis, storage, and eventual fitness impacts (Gershenzon, 1994). Increasing production of many – perhaps all – defensive compounds in response to attack must result in increasing demand for carbon. Carbohydrates support the production of plant phenolics, many of which play a role in defense and can easily exceed >25% of plant dry mass, via the shikimic acid and phenylpropanoid pathways (Harding et al., 2009). Plant terpenes are also carbon-intensive defenses; they are, in general, more expensive to produce per gram than other classes of plant defenses, with additional costs incurred for storage (Gershenzon, 1994). The carbon cost of alkaloids is also high; for instance, nicotine requires 3.62 g of glucose per gram produced, representing about 17% of a tobacco plant’s net carbon gain (Wink and Roberts, 1998). Even glucosinolate production can increase the demand for photosynthate by 15% or more (Bekaert et al., 2012). It is clear that ready access to carbon supplies is essential for plant defense as well as for growth.

If the local demand for carbon exceeds the local ability to fix it from CO_2_, demand must be met via translocation from other sources. However, sites of carbon assimilation and storage (sources) can be quite distant from tissues where utilization creates demand (sinks), requiring translocation over considerable distances. Vascular connections integrate these sites, facilitating carbon transport from sources to sinks to manage the metabolism, growth, and differentiation of shoots, stems, roots, and reproductive structures (Kramer and Kozlowski, 1979). Sucrose is the main transport sugar in most plants, where it may represent >95% of transported carbon (Thorne and Rainbird, 1983; Kozlowski, 1992). Other species transport carbon primarily as larger oligosaccharides and sugar alcohols such as sorbitol. Reducing sugars are rarely transported, if at all (Dinant et al., 2010). The phloem is the major route of carbon transport, although there is evidence for limited xylem transport of carbohydrates in *Acer*, *Betula*, *Salix*, *Vitis*, and *Populus* (Millard and Grelet, 2010). Phloem sap also carries certain defensive compounds, such as alkaloids, flavonoids, and glucosinolates (Turgeon and Wolf, 2009; Wink and Roberts, 1998), as well as information signals such as proteins, RNA, calcium ions, and pressure and/or electrical waves (De Swaef et al., 2013). These substances move en masse in phloem sieve elements along a concentration gradient at rates of 10 cm to 1 m per h, depending on the mechanisms of loading and unloading carbohydrates to phloem sieve tubes (Kozlowski, 1992).

Whether redirecting current photosynthate from sources or reallocating from storage, patterns of carbon movement are constrained by plant phyllotaxy and the arrangement of vascular connections (Jones et al., 1993; Orians, 2005). Even among directly connected plant tissues transport can be affected by tissue size and distance, disrupted by damage to the phloem (e.g., as in girdling) and interrupted at branch points, for example in *Populus* (Arnold et al., 2004; Appel et al., 2012a).

## CARBON SOURCES

Carbohydrates originate in photosynthetic tissues such as mature leaves or storage organs such as roots and stems. These are called “sources.” The usual life history for a leaf is that it begins as a sink, dependent on sources for carbohydrate supply. As it matures it transitions from sink to mixed sink and source, to entirely source (exporting CHO). Source leaves may export some or all of their CHOs to storage as well as to sites actively demanding them. Export from sources – from current photosynthesis or from storage – supports the growth and defense of remote tissues and may also increase the efficiency of photosynthetic processes in source leaves by reducing product inhibition (Millard et al., 2007; Ayre, 2011). As for their own defense, mature leaves have age-related limitations; they may tap into their own photosynthate but do not revert to importing sinks. For example, despite the fact that the wound hormone jasmonic acid rapidly reduces photosynthetic electron transport and gas exchange (Nabity et al., 2013), mature source leaves of poplar trees can sustain some induced phenolic synthesis using what is left of their own carbon gain.

Export requires loading sucrose into phloem sieve elements from symplastic or apoplastic compartments via active transport. Apoplastic loading, involving the pumping of extracellular sucrose into phloem cells, is most common and may include sugar alcohols (Sauer, 2007). This is facilitated by the sucrose transporters (SUT), sucrose carriers (SUC), or more generally the monosaccharide transporters, which are driven by negative plant potentials. Chen et al. (2012) recently showed that apoplastic sucrose loading in *Arabidopsis* is a two-step process that requires the activity of two SWEET sucrose efflux transporters, AtSWEET11 and 12. This type of active phloem loading and phloem transport can be disrupted by loss-of-function mutations or pharmacological inhibitors of these transporters (e.g., Geiger, 2011; Geiger and Savonick, 1975; Turgeon and Gowan, 1990). A second type of active loading occurs symplastically via the polymer trapping mechanism and involves interconnected intermediary cells and the production of raffinose-type oligosaccharides (Fu et al., 2011).

These active loading mechanisms are common in herbaceous plants, which can concentrate sucrose to concentrations as high as 1 M and generate relatively high phloem pressures (Fu et al., 2011; Geiger, 2011). On the other hand, phloem loading also occurs passively by diffusion, sometimes facilitated by membrane proteins called sucrose facilitators (SUF; as in Ayre, 2011), and this is seemingly restricted to woody species exhibiting lower phloem pressures (Gamalei, 1989; Rennie and Turgeon, 2009). It has been proposed that these differences in loading mechanisms, and therefore phloem pressures, among plants may influence patterns of carbon partitioning, defense, and CO_2_-responses (Korner et al., 1995; Long et al., 2006; Turgeon, 2010; Fu et al., 2011).

## CARBON SINKS

Sinks are tissues to which photosynthate is drawn from sources. Sink strength, a tissue’s relative ability to draw in and unload photosynthate, is determined by the activities of enzymes such as sucrose-cleaving invertases and H^+^ coupled transporters as well as by proximity to exporting sources and competing sinks. Cell wall invertase (CWI), an enzyme that is ionically bound to plant cell walls, facilitates phloem unloading at sink tissues by cleaving sucrose into fructose and glucose (Sturm and Tang, 1999). Sugar transporters also contribute to carbon unloading at sinks. The activity of sink enzymes is tightly regulated by transcriptional, post-transcriptional, and post-translational mechanisms (Liesche et al., 2011) which are, in turn, affected by mechanical damage, grazing, infection, galling, all classes of plant hormones, and the development of floral organs, fruits, and seeds, among other tissue types (Roitsch and González, 2004).

Young, developing leaves, fruits, and roots are sinks, and do not possess the resources required to support their own growth and development. Because their demand for sugars is unmet by local supply, carbohydrates are imported. Phloem unloading at these sites may be symplastic or apoplastic (Wolswinkel, 1985), with or against a concentration gradient (Fondy and Geiger, 1982). Tissues may be meristematic, elongation, respiratory, or storage sinks depending on their primary “need” (Chen et al., 2001). Leaves, stems, and coarse roots may transition to source tissues capable of exporting carbohydrates as they age, but developing fine roots, fruits, flowers, seeds, or nectories are sinks their entire lives (e.g., Vassilyev, 2010).

## COMPETITION AMONG SINKS

Since sink strength is defined as a tissue’s ability to import photosynthate relative to other tissues, it is assumed that sink tissues may compete for available carbohydrates. Plant ontogeny is characterized by a predictable sequence of shifting sink priorities, from germination through reproduction. For example, the roots and stems of many tree species alternate sink status to facilitate CHO flow to growing roots in the early season before bud break, then to emerging buds, and back to storage sites in roots and stems in the fall (Shiroya et al., 1966; Ziemer, 1971; Teskey and Hinckley, 1981; Nguyen et al., 1990). Meristems, flowers, fruits, or roots may be the strongest sinks at various times during plant development. Evidence for sink competition can be seen in the impacts of the common horticultural practice of removing competing sinks such as buds, flowers, or fruits (e.g., Smith and Holzapfel, 2009). This redirects CHO flow to remaining sink modules without altering the dry matter production of the plant as a whole (Ho, 1988; Berninger et al., 2000). Herbivores, such as larger browsers, alter CHO flow patterns when they remove source and sink tissues unevenly.

Sinks created by insect or pathogen attack are also potential competitors for photosynthate, both with the plant’s normal sinks and each other. Sink-driven competition is best characterized for galls elicited by aphids. Inbar et al. (1995) found competitive interactions among galls of several aphid species on pistachio leaves. Larson and Whitham (1997) demonstrated competition between aphid galls and meristematic sinks on cottonwood trees in which the number of buds drawing resources negatively impacted gall success.

The development of strong, competing sinks is the basis of the negative impacts on wheat and rice growth by the galling pests, Hessian fly and rice gall midge (Harris et al., 2006; Williams et al., 2011). Sink creation is widespread among galling insect species (Bronner, 1992) and has been shown for free living insect herbivores as well; it should be regarded as an important element of the impact insects can have on their host plants as well as on each other (Baldwin and Preston, 1999; Allison and Schultz, 2005).

Bacterial and fungal pathogens and symbionts also create sinks at the point of infection (Hatcher, 1995; Schaarschmidt et al., 2006). They may compete for limited resources, drawing them away from processes such as grain filling in wheat (Bancal et al., 2012). Microrganisms may also interact with defense responses in more complex ways; for example, foliar wound responses in *Medicago* induce plant-wide increases in CWIs which have a positive effect on mycorrhization (Landgraf et al., 2012). Microrganisms should be factored into attempts to understand resource partitioning when plants response to grazers.

Induced sink strength also can be “cooperative”; for instance, poplar leaves immediately adjacent to induced leaves also exhibit enhanced carbon import and defense responses when sink strength in the neighboring leaf is elicited (Arnold et al., 2004). And galls of the aphid *Hormaphis hamamelidis* (Homoptera: Hormaphididae), which form strong sinks on witch hazel leaves, acquire even more resources when located near fruits, which are also strong sinks (Rehill, 2001).

Some sink-inducing pests appear to have little or no impact on others. For example, reciprocal interactions between root-feeding nematodes and leaf-feeding caterpillars on tobacco was found to be independent of sink strength (Kaplan et al., 2011), and some of the galling aphid species on pistachio leaves do not exhibit negative impacts on each other (Inbar et al., 1995). Browser impacts on plants can create regrowth that is more or less suitable for insect herbivores, depending on the system and time of year (Baldwin and Schmelz, 1994; Nykänen and Koricheva, 2004; Iqbal et al., 2012).

## RESOURCES: NITROGEN

Nitrogen is often a growth-limiting nutrient for plants, and is required for the production of all plant defenses against herbivores and pathogens either as a component of biosynthetic enzymes or of the defenses themselves, such as proteinase inhibitors, chitinases, alkaloids, and glucosinolates.

Inorganic nitrogen as nitrate and ammonium is taken up by roots and is incorporated into amino acids (glutamine and glutamate) used to synthesize other amino acids and nitrogenous compounds. This takes place in root or shoot, depending on the molecular species of nitrogen taken up and the carbon/nitrogen balance of the plant (Andrews et al., 1986; Marschner, 1995; Kruse et al., 2002). These synthesized amino acids move to sink organs (e.g., growing roots and leaves, flowers, and seeds) that are largely dependent on long-distance supply of nitrogen (Pate, 1973; Pate et al., 1983) *via* xylem. Xylem-to-phloem and phloem-to-xylem transfer occurs in the root and the stem. Some nitrate is reduced in the leaves and exported to the root and shoot apex via phloem, producing amino acid cycling (Okumoto and Pilot, 2011). Plant species that produce nitrogen-intensive defense compounds like alkaloids or glucosinolates may transport those products over long distances from points of synthesis (often roots) to points of action (e.g., Baldwin et al., 1994; Chen et al., 2001).

Plants may store nitrogen as protein for protracted periods. Vegetative storage proteins can be assembled rapidly and broken down rapidly for transport to N-demanding sinks via phloem (Staswick, 1989). Amino acid nitrogen may be stored for months or years in stems, roots or foliage as vegetative storage proteins or in active proteins such as RuBisCO (Millard and Grelet, 2010). In the spring developing leaves are strong sinks for stored nitrogen; nitrogen reserves stored overwinter in the bark and wood support up to 90% of new leaf growth in trees (Millard, 1989; Welter, 1989; Millard and Proe, 1991; Sagisaka, 1993; Stepien et al., 1994; Frak et al., 2002; Millard and Grelet, 2010). Changes in N allocation occur naturally in response to the seasons and plant phenology (Millard et al., 2001; Masclaux-Daubresse et al., 2010; Millard and Grelet, 2010); the allocation to bark storage proteins occurs in response waning daylength and colder temperatures (see Millard and Grelet, 2010). Stored nitrogen is allocated among tissues and organs on a competitive basis. For example, removing expanding buds of *Betula pubescens* being supplied by stored proteins redirects allocation to remaining buds or other tissues (Lehtilä et al., 2000; Millard and Grelet, 2010). Releasing stored nitrogen also allows plants to cope with stress and herbivore attack, in part by supporting regrowth or compensatory growth and is an important element of a plant’s ability to tolerate attack (Millard and Grelet, 2010).

The distribution of nitrogenous compounds *via* xylem transport and xylem-phloem transfers is complexly regulated, and may differ with type of compound, plant species, and function of the nitrogenous compounds (storage, defense, growth). N-demanding tissues appear capable of signaling their need for nitrogen to distant sources (Kohl et al., 2012). Movement of solutes in xylem is related to transpiration rates and may be regulated by hormones such as abscisic acid (ABA) (Desclos et al., 2008). Since smaller, growing leaves have relatively low transpiration rates but a high nitrogen demand, other factors must regulate the flow of nitrogenous compounds to these leaves. Large families of genes encoding amino acid, nitrate, alkaloid, and glucosinolate transporters have been identified (Chen et al., 2001; Fan et al., 2009; Shitan et al., 2009; Nour-Eldin et al., 2012). These transporters are involved in loading their substrates into xylem, unloading at sinks, and transferring substrates between xylem and phloem (Gessler et al., 2003). The activities of many of these transporters have not been characterized, and a coherent, detailed model of the regulation of transport via xylem has not yet emerged.

## TYPES OF RESOURCE REALLOCATIONS IN PLANT DEFENSE RESPONSES

### WOUND-INDUCED SINKS AND RESOURCE IMPORT

Both herbivore and pathogen attack begin as localized events. Both also suppress photosynthesis (Creelman and Mullet, 1997; Beltrano et al., 1998; Hristova and Popova, 2002; Izaguirre et al., 2003; Kocal et al., 2008; Schwachtje and Baldwin, 2008; Gomez et al., 2010). Therefore, the ability of individual plant tissues to accumulate defensive substances in response to attack depends on a supply of photosynthate, nitrogen-containing compounds, and other building blocks. This demand can be met by increased sink strength at the attack site, drawing materials from distant sources. The strong connection between regulation of source/sink relations and pathogen success makes sink-regulating apoplastic invertases a key part of plant defense against microbes, too (Berger et al., 2007). It seems clear that long-distance transport of resources is a component of plant defense responses to insects and pathogens (Truernit and Sauer, 1995; Arnold and Schultz, 2002; Roitsch et al., 2003; Roitsch and González, 2004; Matyssek et al., 2005; Appel et al., 2012a, b).

A dramatic increase in local sink strength can be triggered by insect attack, microbial infection, mechanical wounding, natural systemic signals, and artificial elicitors in tomato (Ohyama and Hirai, 1999), carrot (Sturm and Chrispeels, 1990), goosefoot (Ehness et al., 1997), pea (Zhang et al., 1996), hybrid poplar trees (Arnold and Schultz, 2002; Arnold et al., 2004; Philippe et al., 2010; Appel et al., 2012a), and oaks (Allison and Schultz, 2005) among others. Increases in sink strength elicited by infection or herbivory enhance the import of phloem contents to those tissues. For example, insect grazing, jasmonic acid, and mechanical damage increased the import of carbohydrates to developing leaves of *Populus* by as much as 400% and contributed to the synthesis of polyphenols (Arnold and Schultz, 2002; Arnold et al., 2004). When CHO import was disrupted by girdling or the removal of nearby source leaves, the production of these substances was reduced or eliminated. Frost and Hunter (2008) found that red oak seedlings increased allocation of ^13^C from roots to caterpillar-grazed foliage and reduced transport to fine roots.

Ferrieri et al. (2013) examined the impact of MeJA on ^11^C flow in *Arabidopsis thaliana* over 2–24 h. In this case, ^11^CHOs were rapidly exported from labeled source leaves and transported to the MeJA-treated sink leaves where they were incorporated into cinnamic acid, a precursor for phenolic biosynthesis. Export to roots also increased at the same time. Ferrieri et al. (2012) also demonstrated that a radioactive glucose, [^18^F]fluoro-2-deoxy-D glucose, was transported toward wounded *Arabidopsis* leaves and incorporated into phenolic glycosides. In these experiments, like those in hybrid poplar, increases in invertase activity and the accumulation of phenolic compounds were observed in young leaves. Interestingly, in *Arabidopsis* phloem flow began within 2 h, before sink tissues exhibited measurable induced CWI activities. In addition, the accumulation of phenolics in leaves was reduced in mutants lacking SUT needed for phloem loading at source tissues (Ferrieri et al., 2012), suggesting that the flow of CHOs toward wounded tissues may be regulated in part by sources as well as by sinks.

Not all tissues can respond to the same extent with increased sink strength. The ability to increase sink strength, like other induced defense responses, wanes with tissue age. For example, poplar tree leaves lose their ability to respond consistently to elicitation with increased CWI activity as they age and become sources (Arnold et al., 2004). Both invertase activity and ^13^C import decrease as poplar branch and leaf lengths increase and the age structure of the leaves on them changes toward older (Appel et al., 2012a). At some point aging source leaves cannot not revert to importing sinks when elicited, although they may slow their rates of CHO export in response to wounding or jasmonate (Nowak and Caldwell, 1984; Welter, 1989; Schwachtje and Baldwin, 2008). The ability of a leaf to respond to elicitation can change dramatically with its sink/source status over a few days. Studies of induced defenses as well as sink strength need to account for the progressive and sometimes rapid transition from sink to source seen in most plant tissues.

These relationships among elicitation, sink strength, and import of materials have been studied mainly in plants whose defense chemistry is dominated by carbohydrates. The impact of sink strength on induced defenses that include significant amounts of nitrogen or other elements is not as clear. Nitrogen transport and partitioning can be altered by treatment with the wound hormone, jasmonic acid (e.g., Beardmore et al., 2000; Rossato et al., 2002; Meuriot et al., 2004). Nicotine, which is 17% nitrogen by mass and requires ~6% of the nitrogen assimilated by roots, is synthesized in the roots of tobacco and transported to leaves via the xylem stream in response to aboveground jasmonic acid (JA), wounding or herbivory (Baldwin et al., 1994). This movement has not, however, been associated with induced sink strength in elicited leaves.

We would expect that movement of any compounds traveling in phloem would be subject to JA/damage/infection-induced sink strength, but compounds moving in xylem would not. Appel et al. (2012a) found that JA treatment of the young leaves failed to change rates of ^15^N transport from roots to leaves in year-old poplar saplings. ^15^N transport from roots also was not disrupted by steam girdling, indicating that the ^15^N label was largely restricted to xylem and independent of source-to-sink phloem flow (Appel et al., 2012a). This was confirmed in larger, 4-year-old trees, using whole branches, longer transport distances, and multiple sampling intervals (Arnold et al., 2004; Appel et al., 2012b). JA treatment led to significantly lower rates of ^15^N import at 24, 48, and 72 h. It is possible that nitrogen from sources other than roots might have reached the elicited leaves (e.g., stems; Welter, 1989; Fan et al., 2009); that nitrogen would not be labeled and hence would not be detected. Also, it is not clear in what form the ^15^N would have been transported. The most common form in poplar would be amino acids, and there is generally little transfer of amino acids from xylem to phloem in that genus (Gessler et al., 2003). The most likely interpretation of these results is that nitrogen acquired by roots is translocated to leaves in a form that is restricted to xylem, at least until it reaches the leaves. On the other hand, the widely-observed increase in alkaloid movement from roots to attacked leaves in the Solanaceae clearly indicates an increase in the loading of nitrogenous compounds into xylem in response to aboveground signals (Baldwin et al., 1994).

Evidence to date suggests that the import of CHOs to wounded plant tissues *via* enhanced sink strength is a common response. However, there is less evidence for sink-driven import of N by wounded tissues in most plants. And as mentioned above, some of the mechanisms likely to determine loading and unloading of nitrogenous compounds for transport may be responsive to stimuli or signals originating at distant (e.g., attacked) locations (Davis et al., 1991). But because many nitrogen- or sulfur-rich molecules travel in xylem, others in phloem, and others still in both/either, it is not possible at this point to develop a general model of the impact of local infection or herbivory on nitrogen transport.

Increased sink strength by definition increases the flow of sugars to the sink site, irrespective of the elicitor or tissue function. Sucrose and glucose are signals, and alter diverse physiological functions, including the synthesis of phenolics (Rolland et al., 2006). It would appear that any strong sink is likely to accumulate phenolics, irrespective of the tissues’s function (Appel et al., 2012b). This is particularly conspicuous when a sink is created, for example by aphids, to draw amino acids or other low-concentration nitrogenous materials, producing an excess of sugars at the sink site. Examples of this phenomenon might include aphid galls (Rehill and Schultz, 2012) and very young, developing leaves, both of which accumulate large amounts of anthocyanins despite an apparent lack of defensive function for these compounds (Gould and Lister, 2005). Similarly, blocking CHO flow often also causes sugars to accumulate at upstream locations, sometimes visible as anthocyanins, a process some have called termed “pseudo-induction” (Arnold et al., 2004; Steele et al., 2006). We suspect that accumulation of phenolics at sinks, especially anthocyanins, and perhaps other sugar-intensive chemicals is not defensive, or if there is a defensive role this is an exaptation following on a need to dispose of excess sugars (Gould and Lewontin, 1979; Rehill and Schultz, 2012; Appel et al., 2012b).

### WOUND-INDUCED EXPORT AND PLANT TOLERANCE

Wounding, JA treatment, or insect attack have been observed to cause a brief increase in the movement of CHOs away from elicited sites (Babst et al., 2005; Schwachtje et al., 2006; Newingham et al., 2007; Thorpe et al., 2007; Babst et al., 2008; Kaplan et al., 2008; Gomez et al., 2010). The interpretation of such results has been similar to that described in the plant pathogen literature, namely that the plant places valuable nutrients out of the reach of attacking herbivores or diseases, preserving resources for later growth and allowing the plant to tolerate herbivory. This has been described as “bunkering” or “sequestering” resources (Babst et al., 2005, 2008; Schwachtje et al., 2006; Newingham et al., 2007; Kaplan et al., 2008; Gomez et al., 2010; Orians et al., 2011). These studies have employed short-lived radioisotopes, most often ^11^C, to track flow within hours after elicitation. For example, Schwachtje et al. (2006) found that in *Nicotiana attenuata*, simulated grazing and the removal of sink leaf tissues both resulted in an increased flow of carbon to root tissues within 5 h. These plants exhibited prolonged flowering and increased seed capsule production later, although remobilization from belowground stores to accomplish this has not been demonstrated. Gomez et al. (2010) observed similar induced CHO export in tomato, showing that ^11^C export rate from labeled source leaves increased from 27% to as much as 36%, 4 h after MeJA exposure or with the addition of regurgitant from the herbivore *Manduca sexta* (Steinbrenner et al., 2011).

The allocation of ^11^C from leaves to roots may require a biological elicitor. ^11^C labeled source leaves normally exhibit a 1:1 apportionment of carbon to shoots vs. roots but following the application of damage + caterpillar regurgitant 75% of the exported ^11^C traveled toward the roots (Gomez et al., 2012). From principle component analyses of metabolite profiles these authors hypothesized decreased starch levels in the elicited leaf, suggesting that the mobilization of this carbon pool supported respiration and defense processes. Babst et al. (2005) reported a similar response in trees, observing that ^11^CHOs were exported from mature source leaves of *Populus tremuloides* and *P. nigra* saplings at an increased rate when the ^11^C-exposed leaf was itself treated with jasmonates. ^11^C was allocated mainly to lower stems and roots within a few hours (also see Bassman and Dickmann, 1985). However, a subsequent experiment in which jasmonate treatments were replaced by insect herbivory showed that acropetal ^11^C transport from grazed LPI7 did occur and increased relative to ungrazed controls. (Babst et al., 2008).

The sequestration studies finding movement of materials away from elicited leaves are not directly comparable with the studies finding a net movement of photosynthate to elicited sites. First, the sequestration studies have all used short-lived radioisotopes and measured transport within minutes or a few (<5) hours of elicitation. Most studies of transport to elicited sites measured transport 12 or more hours after elicitation. Second, sequestration studies have elicited and focused on movement of materials from older leaves, which are partly or entirely sources, while the long-term movement studies focused narrowly on very young leaves that were known to be entirely or nearly entirely sinks. Such young leaves are not yet exporting materials, which is why they need to import for defense. Older leaves (e.g., on poplar trees), can be induced to increase phenolic synthesis but they cannot elevate sink strength, so they must use their own photosynthate for defense.

More recently, Ferrieri et al. (2013) found that ^11^CO2 fixation and ^11^C-photosynthate export from a labeled source leaf increased to both roots and leaves by 2 h after MeJA treatment of young *Arabidopsis* leaves. By 24 h, resource allocation toward roots returned to control levels, while allocation to the young leaves increased. These movements were associated with altered CWI activities in both leaves and roots, implicating sink-source regulation as a key allocation mechanism. The emerging picture is one of finely-tuned resource reallocation to both belowground and aboveground sinks early, followed by preferential allocation to aboveground sinks later. Interestingly, these allocation responses by leaves were abolished when roots were chilled, suggesting that roots control aboveground allocation decisions *via* long-distance signaling (Ferrieri et al., 2013).

The role of roots and their connection in source-sink relationships and defensive responses may be influenced significantly by the rhizosphere environment and interactions with other plants. Mycorrhizal infection elevates invertase activity and sink strength in roots (Schaarschmidt et al., 2006) and the mycorrhizae provide connections with through which photosynthate and signals are transferred to other plants (Simard, 2009; Song et al., 2010). Presumably the same source-sink interactions that manage translocation within a plant also govern transfers between plants (Simard, 2009). In communities with such mycorrhizal connections the sink strength of nearby plants ought to compete with sinks of their neighbors, with community-level consequences.

Additional resources drawn to root sinks also are exuded into the rhizosphere, where they influence nutrient availability and the composition and function of the microbiome there (Walker et al., 2003; Frost and Hunter, 2008). Marschner (1995) estimated that up to 21% of all photosynthetically fixed carbon is transferred to the rhizosphere this way. One can hypothesize a role for roots in directing traffic among competing sinks within the same plant and among connected plants in a community, a view somewhat convergent on Darwin’s (1880) view of roots as the plant’s organizing center (or “brain”).

Remobilization of nitrogen away from sites of fungal or bacterial infection is commonplace and usually interpreted as an attempt by the plant to deprive the pathogen of necessary nutrients (Pageau et al., 2006; Berger et al., 2007). Whether plants do this in response to insect attack has not received as much attention. Newingham et al. (2007) showed N export away from roots of *Centaurea maculosa* attacked by an insect, *Agapeta zoegana*. Infested plants shifted N flow to shoots, translocating almost twice as much N to the shoot even as root grazing reduced total N uptake by 30–50%. These authors hypothesized that this response helps *C. maculosa* tolerate this herbivore, maintaining biomass and a perhaps even reproductive output, despite the loss of roots. Gomez et al. (2010) found that treatment with the wound hormone MeJA accelerated export of [^13^N] amino acids from labeled tomato leaves for a brief time after treatment. Increased transport of amino acids glutamate, glutamine, and glycine was described as a “⋯strategy to safeguard valuable resources by storing them in distant tissues away from folivores” (Steinbrenner et al., 2011; see Hanik et al., 2010). Generally, jasmonate, which is produced and accumulates at sites of wounding, herbivory, and other stresses, stimulates nitrogen remobilization by accelerating senescence (Rossato et al., 2002; Desclos et al., 2008).

On the other hand, Millard et al. (2001) and Millett et al. (2005) found no effect of browsing on remobilization of nitrogen away from birch leaves. Grazing often draws nitrogen from increased root uptake or storage to attacked leaves in grasses (Thornton and Millard, 2006, but see Redak and Capinera, 1994). Moreira et al. (2012) found increased nitrogen in phloem apparently traveling toward MeJA-treated foliage (needles) in pine saplings. All of these studies were done on much longer time scales than any of the sequestration studies. It appears that research to date does not permit a clear evaluation of the idea that herbivory induces sequestration or that sink-facilitated import functions for nitrogen resources.

Together, these studies show that some species can transport some resources away from sites of herbivory over a brief period following attack. Removing important nutrients from the reach of immobile plant pathogens, whose rapid growth depends on immediate access to nearby resources, may help limit the attack and protect the plant. But we wonder whether these short-term responses would have any impact on insects, which may feed on a leaf for only a few minutes before moving on and for whom carbon is in excess. Removing nitrogen from a leaf may be more effective, but the evidence for sequestering nitrogen is weaker than for carbon, and mixed for responses to insects compared with responses to pathogens. Resources translocated to roots may be stored there, recycled aboveground, sent to mycorrhizae, exuded into soil, and may even wind up in nearby plants. Until the eventual fate of these resources is determined, it is not possible to know whether they are recovered from roots and used for regrowth or reproduction following attack.

## RESOURCES: TO ADVANCE OR WITHDRAW IN THE FACE OF ATTACK?

The evidence suggests that plants employ both strategies in response to wounding. Resource flow can increase, stop, or reverse within hours in response to attack or elicitation (Kleiner et al., 1999; Arnold and Schultz, 2002; Babst et al., 2005; Schwachtje et al., 2006; Newingham et al., 2007; Babst et al., 2008; Frost and Hunter, 2008; Gomez et al., 2010). And the view that moving photosynthate belowground or to sites of attack as evidence of mutually exclusive strategies – to grow or defend, resist or tolerate – is certainly an oversimplification.

The collection of diverse experiments conducting over the past decade reveal a few consistent trends. Attack by both insects and pathogens clearly alters the movement of resources among plant modules. Further, it appears that changes in rate or direction of the movement of photosynthate are under the control of sink-source interactions, probably involving inducible invertases and other phloem loading/unloading systems. Activities of some of these sink-creating systems are regulated by infection and/or herbivory, but more work is needed to identify eliciting mechanisms. The movement of materials traveling in xylem rather than phloem is influenced by transpiration, but other factors that probably direct these movements have not been characterized. Differences in transport stream – phloem vs. xylem – produce differences in resource distribution. Factors like branching, orthostichy, and differential sink strength that can change the course of carbon movement in phloem apparently have little or no impact on xylem-transported materials like peptides and alkaloids. While comparative studies have yet to be done we would expect this to produce more heterogeneity in the distribution of carbon and carbon-dependent defenses than in the distribution of nitrogen and its dependent defenses. This heterogeneity, which is widely observed, may itself provide a kind of resistance to herbivores (Whitham, 1982; Schultz, 1983). Competition among sinks could influence the ability of a plant organ or module to respond effectively to attack. The root is a powerful and influential sink against which all other sinks – irrespective of type or source – must compete, and through which resources for defense may pass before reallocation above ground. We have evidence that the root’s influence may extend beyond its sink status, to regulate sink-source interactions aboveground. Travel of materials to many – perhaps all – sinks, including the rhizosphere and other connected plants, passes through the root, which may function as a manifold, directing traffic.

Significant gaps in our knowledge remain, however, and it is increasingly difficult to compare individual studies because of differences in experimental design. Recent studies have employed different herbivores or artificial elicitors, with significant discrepancies in the amount of wounding, the number of treatments, the delay between treatments and observations, and the duration and frequency of those observations. They have also tracked resource flow among different plant modules, of different sink/source status, separated by varying distances, using short or long-lived isotope tracers. Seemingly minor variations in experimental design often generate contrasting results. As a result we are generally unable to predict the directionality of resource flow in all but a few very specific cases.

We presume that resource reallocation is advantageous to plants. However, research on resource translocation in response to attack by pathogens or herbivores has tended to focus narrowly on physiological and biochemical mechanisms; considerably more work is needed at the whole-plant level to determine which, if any, of these fascinating plant behaviors actually influence plant fitness.

To date no clear, convincing experimental evidence has yet linked induced resource transport to or away from attacked tissues to plant fitness. To characterize any response as defensive, it must preserve or enhance plant fitness. The only induced resource (not defense) translocation response we have discussed here that might actually protect a plant *via* a relatively immediate impact on insect feeding would be short-term increases in carbon or nitrogen import to elicited tissues in support of defense production (e.g., Ferrieri et al., 2012). Changes in defense chemistry fueled by this rapid import could theoretically influence insect behavior and reduce damage to the plant, either directly or indirectly by increasing the effectiveness of predators and parasites (Schultz, 1983). The suppression of insect growth can be a meaningful measure of “defense” provided the impact on the insect can be shown to benefit the plant. For example, it is well established that the export of resources from pathogen infection sites generally preserves plant fitness by inhibiting the spread of the infection. The best-supported case in which source-sink relationship contributes to plant resistance is probably competition between meristem sinks and aphid galls on cottonwood (Larson and Whitham, 1997; Compson et al., 2011). A study indicating that transporting photosynthate to roots preserved and extended reproduction later in *N. attenuata* (Schwachtje et al., 2006) also comes close, but is subject to alternative interpretations. Strictly speaking, assessing the impact of responses on plant fitness can and probably should require at least a two-generation measurement. In general, well-integrated manipulative experiments that change resource movement through time, track the movements of individual molecules over extended periods, and show a net fitness advantage to the plant are needed to reach meaningful conclusions.

## Conflict of Interest Statement

The authors declare that the research was conducted in the absence of any commercial or financial relationships that could be construed as a potential conflict of interest.
